# Publisher Correction: Intercalation events visualized in single microcrystals of graphite

**DOI:** 10.1038/s41467-018-02957-y

**Published:** 2018-02-19

**Authors:** Edward R. White, Jared J. Lodico, B. C. Regan

**Affiliations:** Department of Physics & Astronomy and California NanoSystems Institute, University of California, Los Angeles, CA 90095 USA

Correction to: *Nature Communications* 10.1038/s41467-017-01787-8, published online 06 December 2017

The Peer Review File associated with this Article was updated shortly after publication to redact confidential comments to the editor.

