# Supplementary material for: Intercalation events visualized in single microcrystals of graphite
Source: Nat Commun. 2017 Dec 6;8:1969. doi: 10.1038/s41467-017-01787-8 (PMC5719043; doi:10.1038/s41467-017-01787-8)
Supplement: Supplementary file 1 — Supplementary Information [file 41467_2017_1787_MOESM1_ESM.pdf]

Supplementary Information

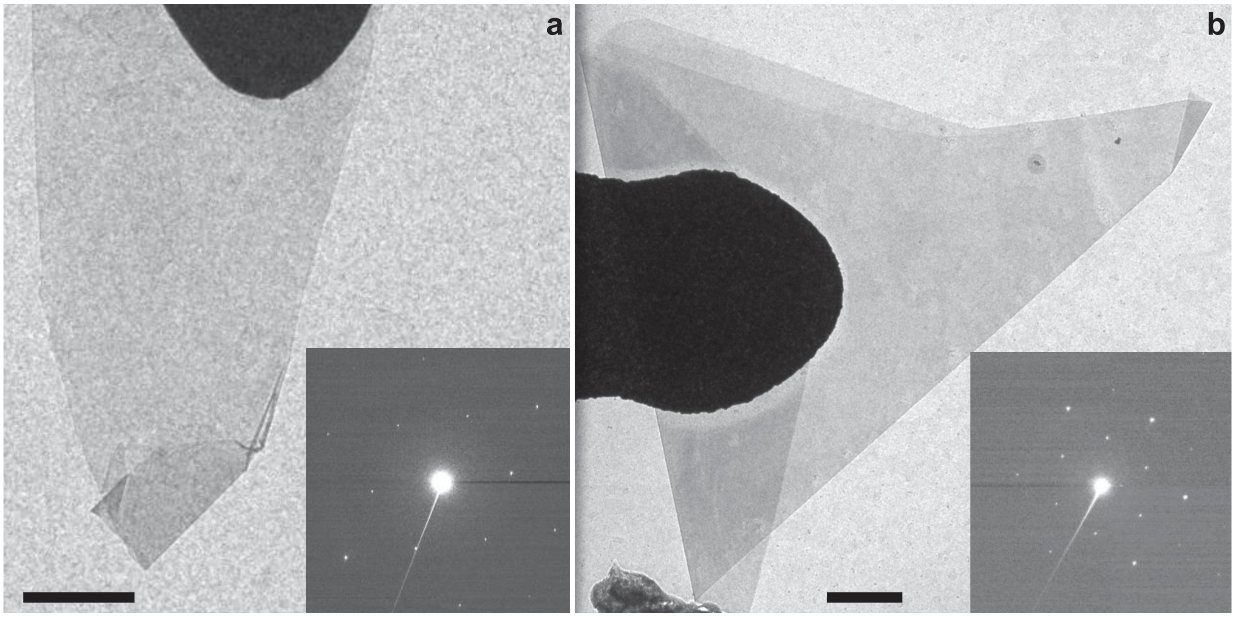

Supplementary Figure 1. Bright field TEM images of graphite flakes prior to the fluid cell construction described in Fig. 1. **a**, Graphite flake from Supplementary Movie 3, Figs 4 and 5, and Supplementary Fig. 5a. **b**, Graphite flake from Supplementary Movie 4, Fig. 6, and Supplementary Fig. 5b. The insets are diffraction patterns acquired with a majority of the flake illuminated either before (**a**) or after (**b**) the construction of the respective fluid cells. Both diffraction patterns show one hexagonal pattern, indicating that the graphite flakes are single crystals. The scale bars are 1 μm.

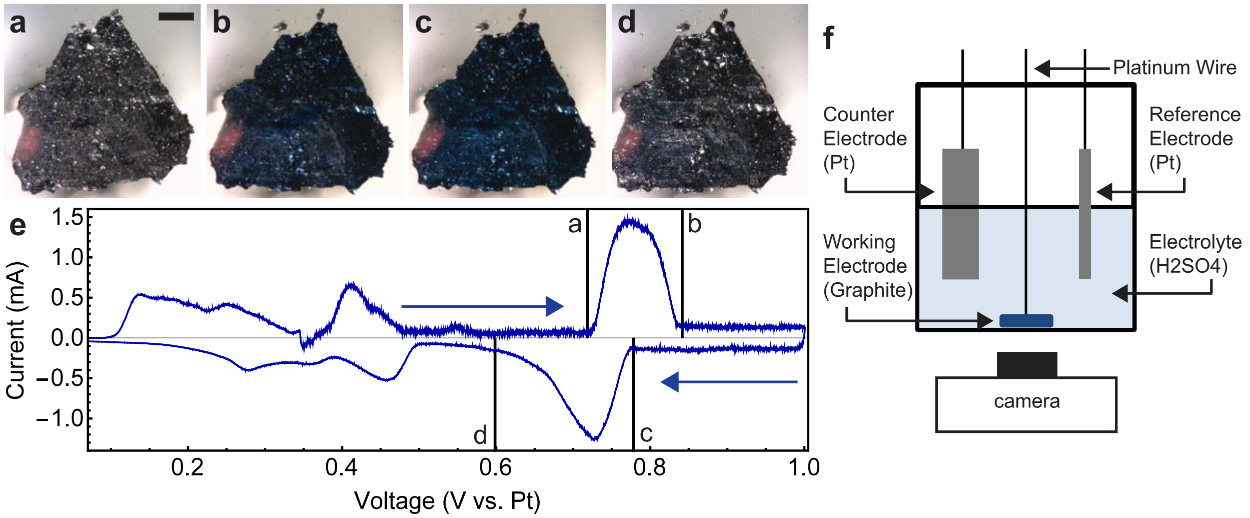

Supplementary Figure 2. The first intercalation cycle of bulk natural graphite (8.11 mg) in sulfuric acid (18 M H_2SO_4, 96%) via cyclic voltammetry. The graphite was fully submerged in the electrolyte and electrically connected through a platinum wire. **a**, **b**, Graphite transition from stage 2→1. **c**, **d,** Graphite transition from stage 1→2. The voltage corresponding to each of the four optical images **a**-**d** is indicated by the black lines in the transport data **e**. Here the potential was ramped at ±0.1 mV s^{-1}. **f,** Schematic of the experimental set-up. See Supplementary Movie 1. The scale bar is 1 mm.

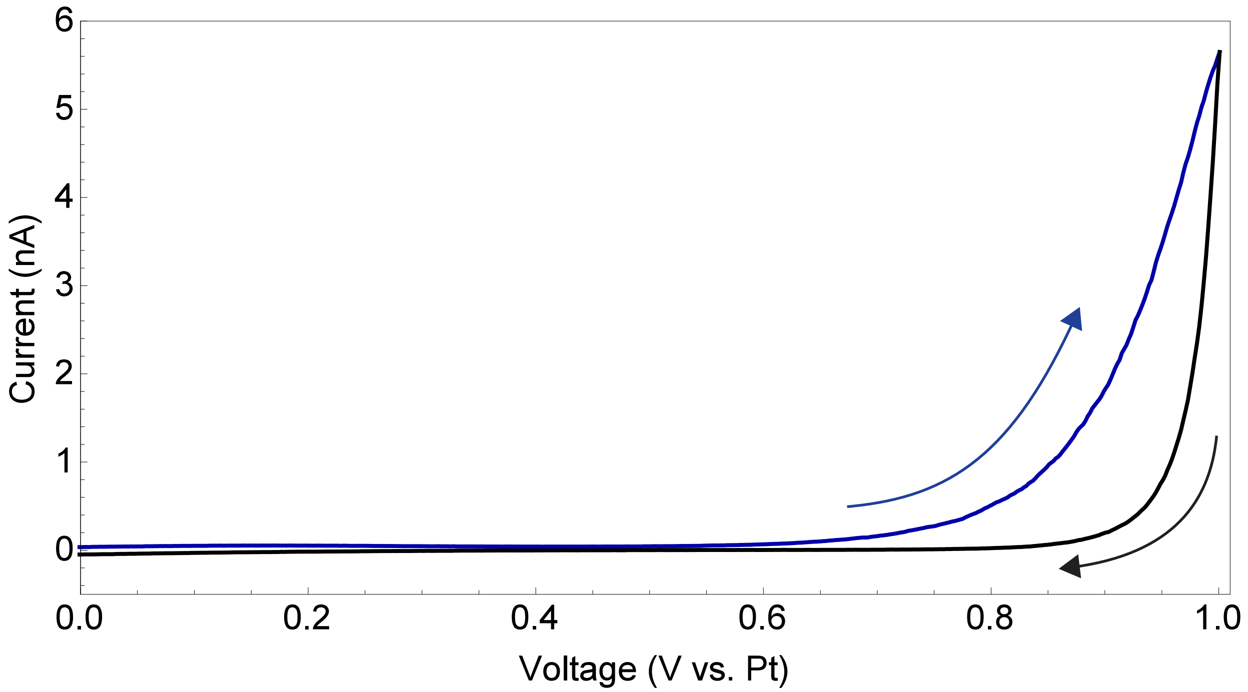

Supplementary Figure 3. Control CV experiment (*ex situ* - no electron beam) using the same type of silicon chip as was used in the *in situ* STEM measurements, but without graphite attached to the working electrode. The working, counter, and pseudo-reference electrodes on the Si chip were all platinum patterned with optical lithography, and were electrically connected by a drop of sulfuric acid. The charging (blue) and discharging (black) currents in the cyclic voltammogram (±20 mV s^{-1}) are attributed to double layer charging. When graphite is transferred to the working electrode the voltammograms show the same background current (see, for example, Figs 2d and 3d and Supplementary Fig. 7) with intercalation events superimposed. This smoothly varying background current is subtracted out to isolate intercalation events (see Supplementary Fig. 4).

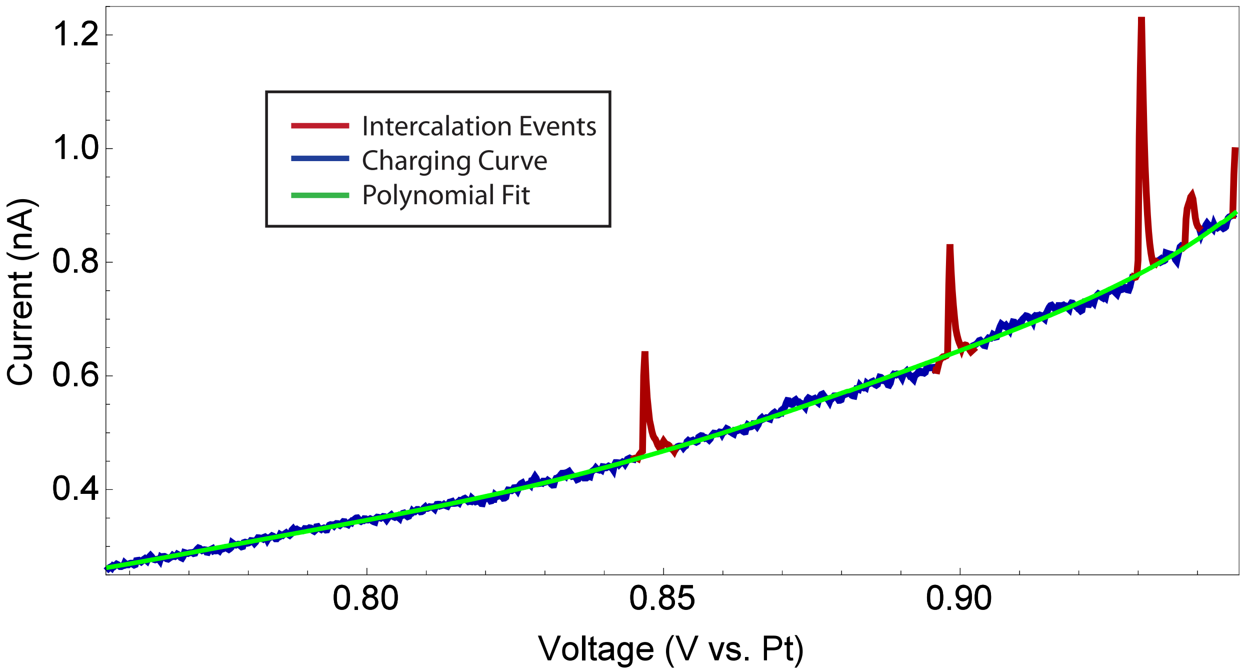

Supplementary Figure 4. A segment of the intercalation curve from Fig. 2d demonstrating the background-subtracting fit procedure. The unprocessed data is the gradual charging trend (blue) and individual intercalation events (red). A 6th order polynomial (green) is used to fit the gradual curve (blue) with the intercalation events (red) masked for the ramped up and down sections of the CV separately. The polynomial fit is then subtracted from the raw electrical transport data to produce a background-subtracted CV (Fig. 2h).

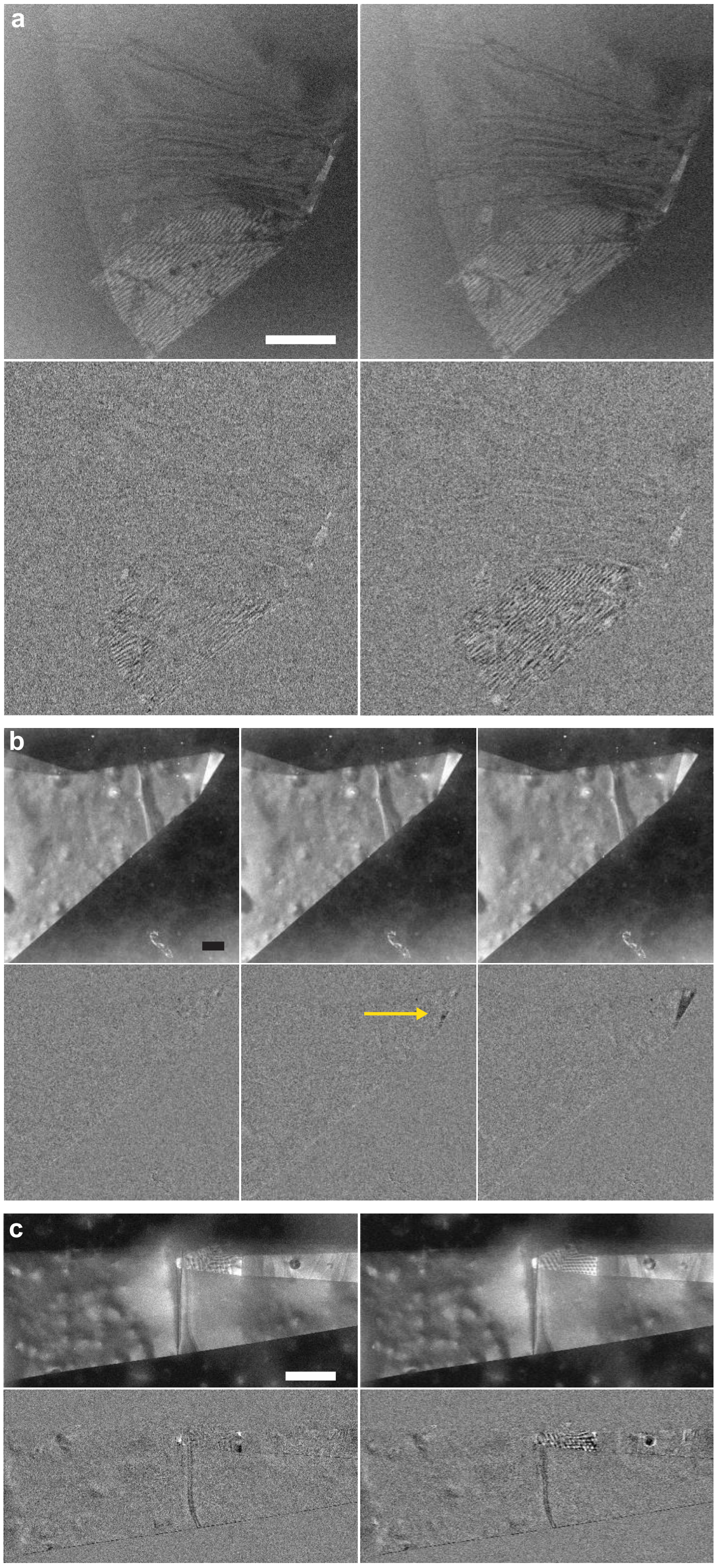

Supplementary Figure 5. a-c, ADF STEM (top) and delta (bottom) images of microcrystal graphite flakes with folds. Moving from left to right the images are sequential frames acquired

before and after the first significant structural changes. The computed delta images = ($frame_n$ – $frame_0$) highlight the changes relative to the first frame of the STEM video. Bright (dark) pixels correspond to an increase (decrease) in ADF intensity. As the potential is increased the graphite electrochemically intercalates, causing the STEM contrast to change. The first structural changes consistently occur in the regions where the graphite has folded onto itself. In **b** an intercalation event, at 1.05 V indicated by the yellow arrow, occurred as the electron beam was scanning over the folded region. As the beam continues to raster over the sample, the following delta image shows the contrast changed over the rest of the fold. The frames in **a** are from Supplementary Movie 2 and Figs 4 and 5. The frames in **b** are from Supplementary Movie 3 and Fig. 7. The scale bars are 500 nm.

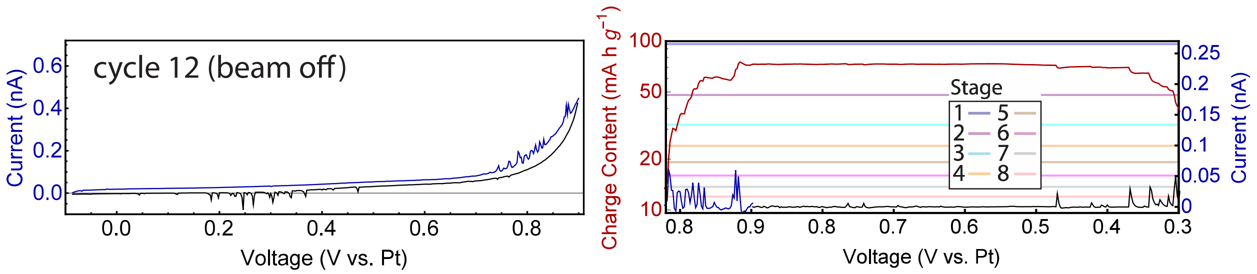

Supplementary Figure 6. Control experiment. CV (left) and background subtracted CV (right) of the graphite from Figs 4 and 5 and Supplementary Fig. 5a performed with the electron beam blanked. These curves are very similar to those generated while the sample was being imaged (shown in the following two supplementary figures), which demonstrates that the imaging electron beam played, at most, a minor role in determining the electrochemical transport.

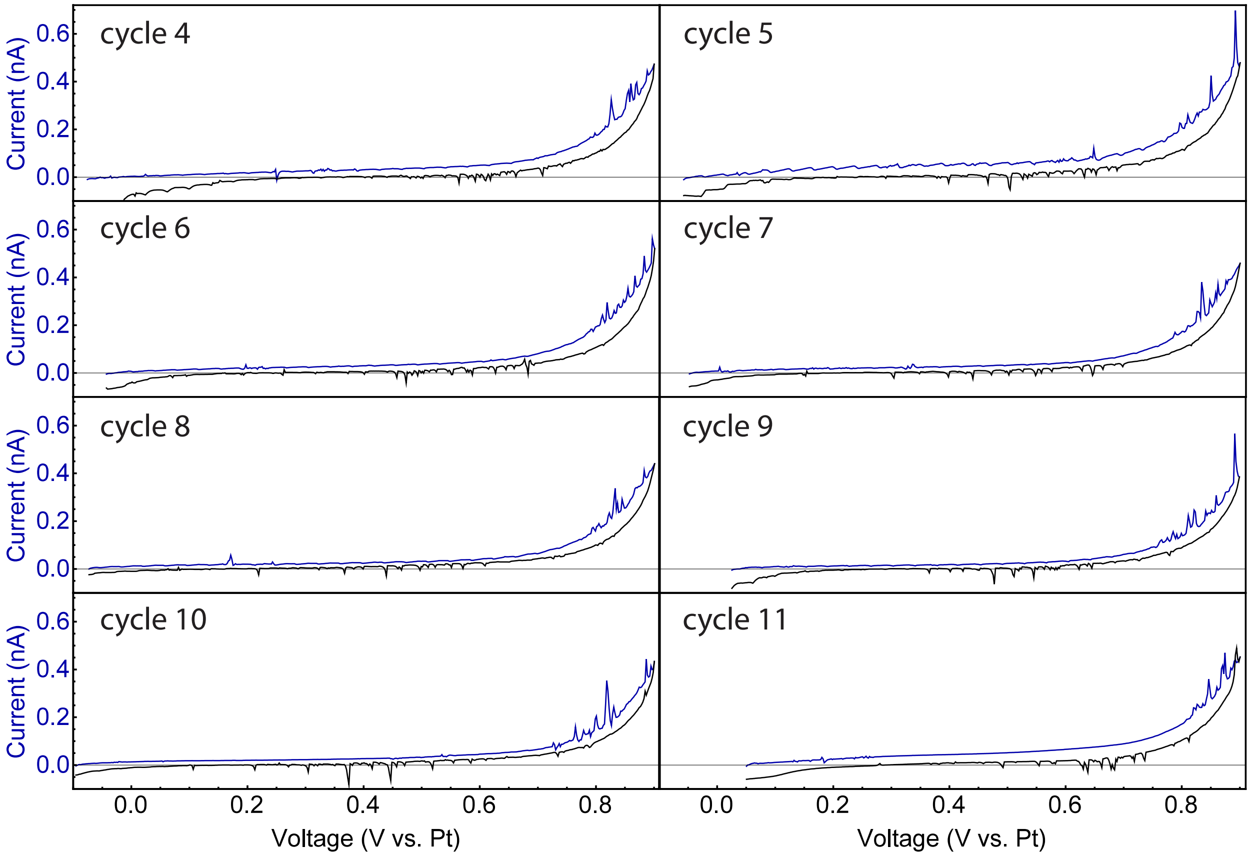

Supplementary Figure 7. Individual CVs of the graphite from Figs 4 and 5 and Supplementary Fig. 5a before the background-subtraction procedure described in Supplementary Fig. 4. The cycle number is indicated on each plot.

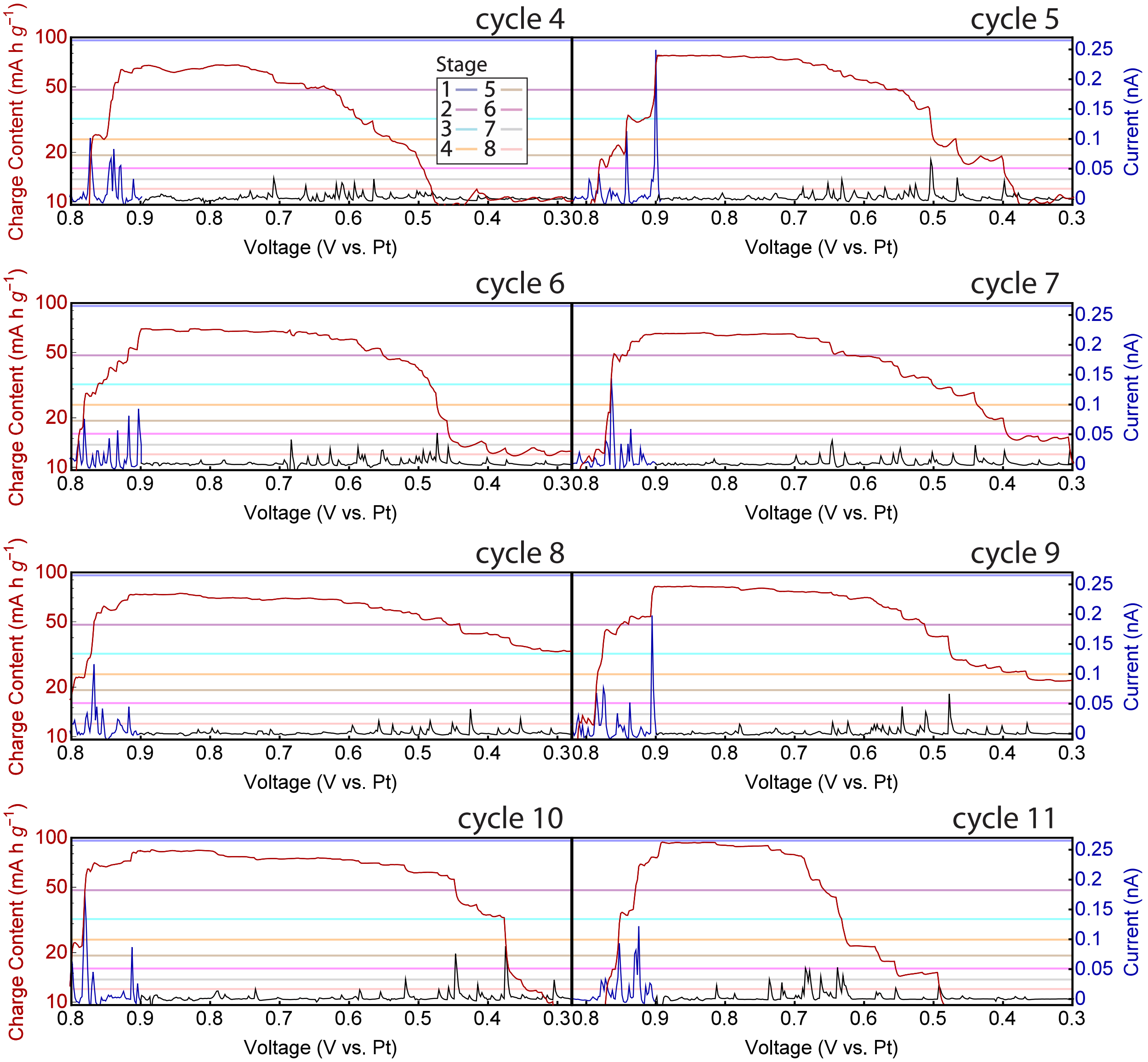

Supplementary Figure 8. Individual background-subtracted cyclic voltammograms of the graphite from Figs 4 and 5 and Supplementary Fig. 5a using the technique described in the caption of Supplementary Fig. 4. The cycle number is indicated above each plot. The blue (black) curves represent positive (negative) current. The red curves give the charge content, calculated by integrating the current. The specific capacities corresponding to stages 1-8 are indicated by the colored lines. Current is plotted on a linear scale and charge content on a logarithmic scale (to better separate high stage numbers).

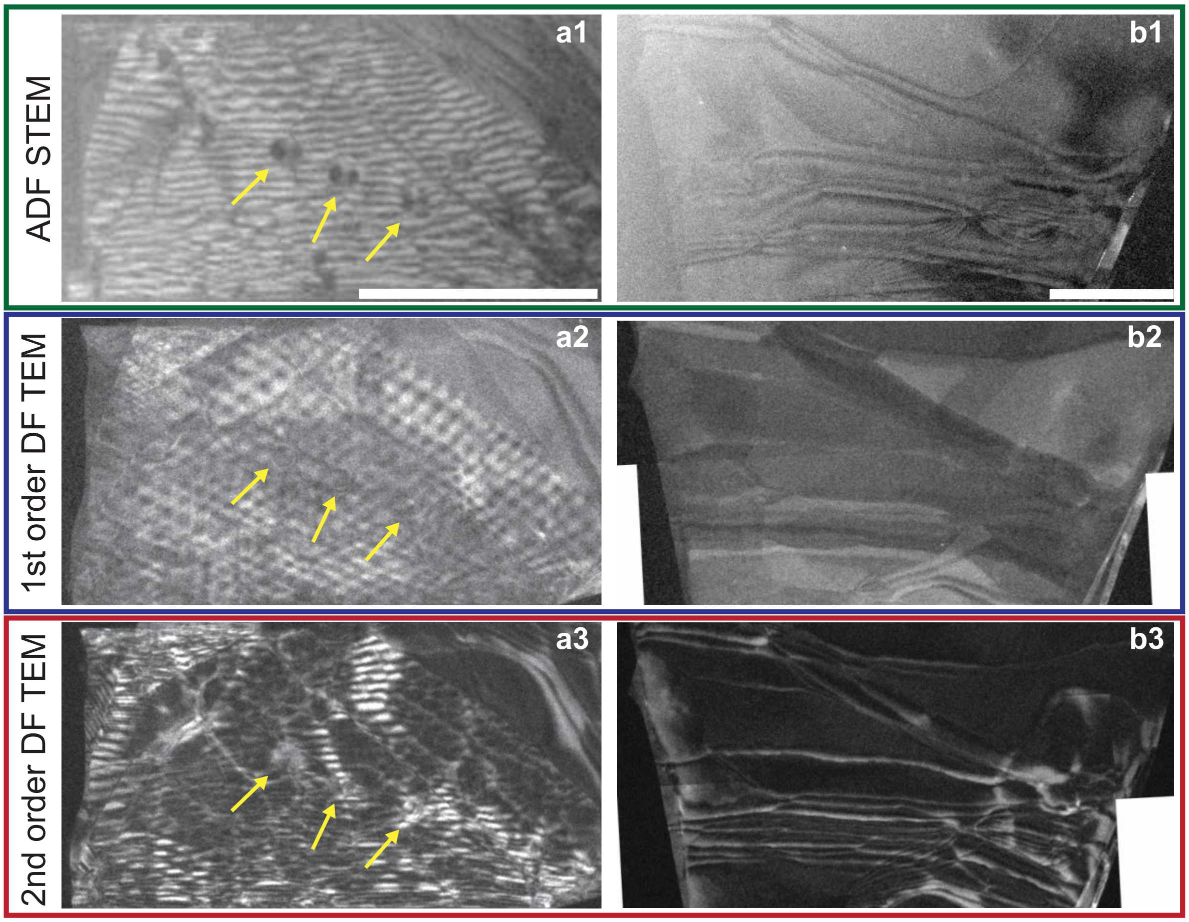

Supplementary Figure 9. ADF STEM, 1st order DF TEM, and 2nd order DF TEM images of graphite. **a1** ADF STEM, **a2** first order dark field TEM, and **a3** second order dark field TEM of the folded region of graphite shown in Figure 4, Supplementary Figures 1a and 5a, and Supplementary Movie 3. The yellow arrows in the dark field TEM images highlight the circular defects also seen in ADF STEM. **b1** ADF STEM, **b2** first order dark field TEM, and **b3** second order dark field TEM of a different region of the same graphite flake, also shown in Figure 5, Supplementary Figures 1a and 5a, and Supplementary Movie 3. The pattern of line defects is consistent across the dark field TEM and ADF STEM images, which show related contrast because of the small convergence angle employed (see Methods). All dark field TEM images were acquired 'dry', prior to construction of the fluid cell, and all ADF STEM images were

acquired ‘wet’, after construction of the fluid cell, but before intercalation. The scale bars are 500 nm.
