# Supplementary material for: Intercalation events visualized in single microcrystals of graphite
Source: Nat Commun. 2017 Dec 6;8:1969. doi: 10.1038/s41467-017-01787-8 (PMC5719043; doi:10.1038/s41467-017-01787-8)
Supplement: Supplementary file 2 — Description of Additional Supplementary Files [file 41467_2017_1787_MOESM2_ESM.pdf]

Description of Additional Supplementary Files

File Name: Supplementary Movie 1

Description: Movie of bulk graphite (8.11 mg) undergoing its first intercalation and deintercalation cycle (last frame shown above). The image video is on the left, while the synchronized cyclic voltammogram (CV) is on the right. Here the potential was ramped at ±0.1 mV s^{-1} and the graphite was imaged at a frame rate of one per 50 seconds. This movie plays at 200 × real time. A summary of this experiment is given in Supplementary Fig. 2.

File Name: Supplementary Movie 2

Description: Movie of single crystal graphite undergoing its first intercalation and deintercalation cycle (last frame shown above). The ADF STEM video is the top left panel. The calculated derivative movie = ($frame_n$ – $frame_{n-1}$) is the top right panel. The synchronized background-subtracted cyclic voltammogram (CV) is the bottom panel. Here the potential was ramped at ±10 mV s^{-1} and graphite was imaged with a frame time of 5.9 seconds. This movie plays at 11.8 × real time. Single still frames are given in Fig. 2.

File Name: Supplementary Movie 3

Description**:** Movie of the structural evolution of graphite during multiple intercalation and deintercalation cycles (last frame shown above). The label displays the cycle number and the total imaging time (hr:min:sec). Each cycle's ADF STEM video is on the left. The calculated derivative movie = ($frame_n$ – $frame_{n-1}$) is on the right. Here the potential was ramped at ±2 mV s^{-1} while the flake was imaged with a frame time of 31.5 seconds. During cycles 5 through 8 various regions of this flake were imaged at higher magnification. The white spots that appear in cycle 9, after approximately 3 hours of imaging, are attributed to beam-induced contamination resulting from the higher magnification imaging. This movie plays at 157.5 × real time. See Figs 4 and 5 for averaged still frames and Supplementary Fig. 7 for CV data.

File Name: Supplementary Movie 4

Description**:** Movie showing the contrast evolution of graphite during the first intercalation cycle (last frame shown above). The ADF STEM video is the top left panel. The calculated derivative movie = ($frame_n$ – $frame_{n-1}$) is the top right panel. The synchronized background-subtracted cyclic voltammogram (CV) is the bottom panel. Here the potential was ramped at ±1 mV s^{-1} while the flake was imaged with a frame time of 20.4 seconds. This movie plays at 81.6 × real time. A summary of this data is given in Fig. 6.

File Name: Supplementary Movie 5

Description**:** Movie showing the contrast evolution of graphite during multiple intercalation and deintercalation cycles (last frame, labeled, shown above). The label displays the cycle number and the total imaging time (hr:min:sec). The ADF STEM video is shown in the left panel. The calculated delta movie = ($frame_n$ – $frame_0$), where "$frame_0$" refers to the first frame of that particular cycle, is shown in the middle panel. The calculated derivative movie = ($frame_n$ – $frame_{n-1}$) is shown in the right panel. Here the potential was ramped at ±2 mV s^{-1} while the flake was imaged with a frame time of 20.4 seconds. This movie plays at 102 × real time.
